# Meteorin-Like Ameliorates β Cell Function by Inhibiting β Cell Apoptosis of and Promoting β Cell Proliferation via Activating the WNT/β-Catenin Pathway

**DOI:** 10.3389/fphar.2021.627147

**Published:** 2021-03-17

**Authors:** Wenchao Hu, Rui Wang, Bei Sun

**Affiliations:** ^1^ Department of Endocrinology, Qilu Hospital (Qingdao), Cheeloo College of Medicine, Shandong University, Qingdao, China; ^2^ Department of Blood Transfusion, Qilu Hospital (Qingdao), Cheeloo College of Medicine, Shandong University, Qingdao, China; ^3^ NHC Key Laboratory of Hormones and Development (Tianjin Medical University), Tianjin Key Laboratory of Metabolic Diseases, Tianjin Medical University Chu Hsien-I Memorial Hospital and Tianjin Institute of Endocrinology, Tianjin, China

**Keywords:** meteorin-like, apoptosis, proliferation, β cell, β-catenin

## Abstract

Meteorin-like (Metrnl) is a newly discovered myokine. Plasma Metrnl is decreased in subjects with newly diagnosed type 2 diabetes (T2D) and correlated with insulin resistance. This study aims to determine the effects of Metrnl on the apoptosis and proliferation of β cell. Mouse insulinoma MIN6 cells were divided into six groups: normal control, low glucose, high glucose, Vehicle, Metrnl, and Dickkopf 1 (DKK1) groups. MIN6 cells in Metrnl group were transfected with recombinant pCDH-Metrnl vector. WNT/β-catenin pathway was inhibited using DKK1. Then the apoptosis of MIN6 cells was detected using flow cytometry and TUNEL labeling. Immunofluorescence of Ki67 or Edu-594 was used to determine the β cell proliferation. db/db mice were confirmed as T2D group. Lentivirus-Metrnl was injected from the caudal vein of db/db mice once every two weeks for two times. High glucose induced the apoptosis of MIN6 cells and elevated expression of caspase 3. In addition, high glucose resulted in reduced β cell proliferation, cell viability, insulin secretion as well as decreased expression of β-catenin and TCF4. Metrnl ameliorated the above effects of high glucose. And the protecting role of Metrnl was inhibited by DKK1. T2D mice showed higher body weight and blood glucose compared with the controls. The β cell apoptosis was increased while the β cell proliferation and WNT/β-catenin pathway were inhibited in T2D mice. Metrnl treatment partly reversed the above changes in T2D mice. Metrnl ameliorates β cell function by inhibiting β cell apoptosis of and promoting β cell proliferation via activating the WNT/β-catenin pathway.

## Introduction

Diabetes mellitus represents a serious threat to global human health and welfare. Type 2 diabetes (T2D) has a characteristic of peripheral insulin resistance and progressive pancreatic β cell dysfunction (Gastaldelli, 2011). β cell dysfunction is primarily caused by the apoptosis of β cells. β cell dysfunction contributes to a reduced capacity to secrete insulin and results in hyperglycemia (Bugliani et al., 2019). The exact mechanism underlying insulin resistance and β cell apoptosis remain unclear.

Skeletal muscle was recently shown to have endocrine functions to produce active metabolites known as myokines (Pedersen and Hojman, 2012). Meteorin-like (Metrnl), a newly discovered myokine, is secreted after acute bouts of exercise and acute cold exposure (Rao et al., 2014). Metrnl up-regulates the expression of genes associated with thermogenesis in brown/beige adipocytes and promotes the production of anti-inflammatory molecules (Rao et al., 2014). Recent investigations have confirmed the association of Metrnl with diabetes. Serum Metrnl levels were found to be lower in T2D patients compared with the healthy controls (Dadmanesh et al., 2018; Lee et al., 2018; El-Ashmawy et al., 2019; Fadaei et al., 2020). In addition, Metrnl administration alleviated the impaired insulin secretion both in palmitate-incubated C2C12 cells and the skeletal muscle of high-fat diet-fed mice (Jung et al., 2018). These results indicate that Metrnl may be involved in the mechanism of maintaining β cell function and diabetes.

This study aims to determine the effects of Metrnl on the apoptosis and proliferation of β cell. Then we also detect the possible mechanism of Metrnl in maintaining β cell function.

## Method

### Cell Culture

MIN6 cells were cultured in Dulbecco's modified eagle medium (DMEM; Invitrogen, Carlsbad, CA, USA) with 10% fetal bovine serum at 37°C in 5% CO_2_ and 88% humidity. Cells were divided into six groups: normal control (NC), low glucose (LG), high glucose (HG), HG + Vehicle, HG + Metrnl, and HG + Metrnl + Dickkopf 1 (DKK1). Min6 cells in LG and HG group were incubated with 5.5mmol/L and 30 mmol/L glucose respectively. To perform the overexpression of Metrnl, MIN6 cells in Metrnl group were transfected with recombinant pCDH-Metrnl vector for 4 h using Lipo6000TM transfection reagent according to the manufacturer’s protocol (RiboBioCo. Ltd, Guangzhou, Guangdong, China), and then were cultured in normal medium for 24 h before the next treatment. The vehicle group was transfected with pCDH blank vector. DKK1 (100ng/mL, Sigma-Aldrich) was utilized to serve as an antagonist of WNT/β-catenin pathway.

### Animals

**FIGURE 1:**
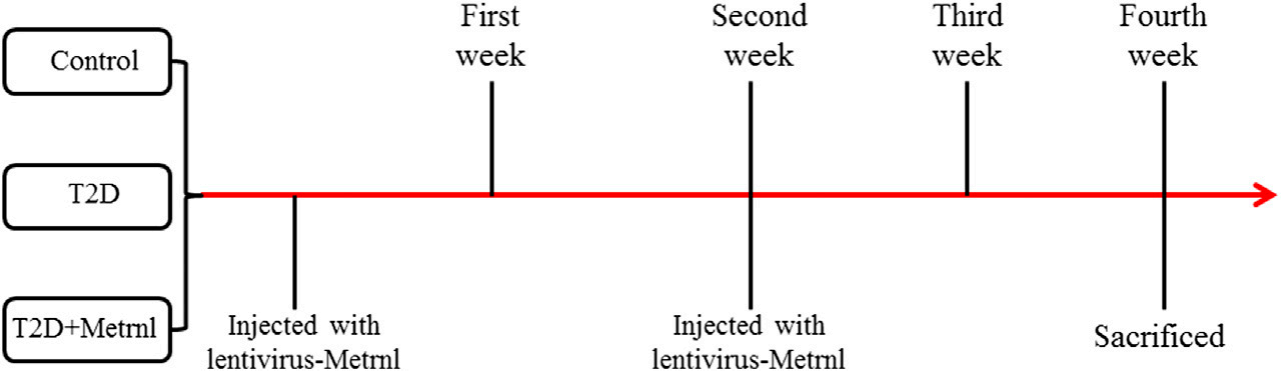
Time axis indicating mice treatments.

Twelve-week-old db/db mice and db/m mice were obtained from GemPharmatech Company of Jiang Su, China and made as the T2D group. After adaptation for one week, the db/db mice were allocated into two groups based on the baseline fasting blood glucose and body weight: T2D and T2D + Metrnl groups. And the db/m mice were assigned to the control group. The mice in T2D + Metrnl group were injected with lentivirus-Metrnl (100ul, 1 × 10^11^ PFU/ml) from the caudal vein once every two weeks for two times (Figure 1). All animals were housed in an environmentally controlled room at 25°C with a 12-h light and 12-h darkness cycle, and fed with a normal diet and with free access to water throughout the experimental period. All animal procedures in this study were approved by the Medical Ethical Committee of our hospital.

### Measurement of Body Weight and Fasting Blood Glucose

The body weight and fasting blood glucose were measured before injection and every week after injection. The fasting blood glucose was detected with venous blood abstracted from the tail using a glucose monitor (Roche, Germany) after fasting for 14 h.

### Pancreas Collection

The mice were sacrificed by decapitation during CO_2_ anesthesia and the pancreas was removed. The pancreases of odd-numbered mice were fixed with 10% neutral buffer formalin solution. And the pancreases of even-numbered mice were stored in liquid nitrogen.

### Quantitative RT-polymerase Chain Reaction (PCR)

Total RNA was isolated from MIN6 cells or pancreatic tissue with the Trizol kit according to the manufacturer's instructions (Invitrogen Life Technologies). The cDNA Synthesis kit (Takara, Otsu, Shiga, Japan) was used to synthesize cDNA. Real-time PCR amplification was performed using the SYBR Green Supermix (Takara, Otsu, Shiga, Japan). The sequences of primers used in the RT-PCR were as follows: Metrnl, 5′-TACGGCCCAACACCTTCTCA-3′(forward) and 5′-GTC​CCG​CAC​CAA​CAG​TCT​TAG​T -3’ (reverse); caspase 3, 5′-AAGATACCGGT GGAGGCTGAC-3′(forward) and 5′-GTT​AAC​GCG​AGT​GAG​AAT​GTG​C-3’ (reverse); transcription factor 4 (TCF4), 5′-CGCTGACAGTCAACGCATCTATG-3′(forward) and 5′-GGA​GGA​TTC​CTG​CTT​GAC​TGT​C-3′ (reverse); β-catenin, 5′-GTTCGCCTTCATTATGGACTGCC-3′(forward) and 5′-ATAGCACCCTGTTCC CGCAAAG-3’ (reverse); β-actin, 5′-CATTGCTGACAGGATGCAGAAGG-3′(forward) and 5′-TGC​TGG​AAG​GTG​GAC​AGT​GAG​G-3’ (reverse). All reactions were performed in triplicate. The target gene relative expression was normalized using β-actin as an internal reference.

### Western Blot

Proteins were extracted from the cells or pancreatic tissue using a RIPA lysis buffer. The protein concentration was quantified by a DC protein kit. Protein samples were carried out with 10% SDS-PAGE, and then transferred to PVDF membranes. The membranes were blocked in 5% milk for 1 h at room temperature, and then were incubated with their primary antibodies overnight at 4°C: Metrnl (1:1,000, Abcam), Cleaved-Caspase 3 (1:2000, Proteintech), TCF4 (1:1,000, Cell Signaling Technology), Non-phospho (Active) β-catenin (1:1,000, Cell Signaling Technology), and β-actin (1:5,000, Proteintech). In the following day, the membranes were incubated with HRP-linked secondary antibodies for 2 h at room temperature. The bands were detected by chemiluminescence and the protein quantification was performed by ImageJ software.

### Flow Cytometry Analysis

An annexin V-FITC/PI apoptosis detection kit (AD10, Dojindo Laboratories, Shanghai, China) was used for apoptotic cell determination with a flow cytometry (ACEA Biosciences, Inc.) following the instruction. The apoptotic cell percentage was defined as the sum of the early and late apoptotic cell percentages.

### TUNEL Labeling

TUNEL assay was used to measure cell apoptosis in MIN6 cells. In brief, cells were seeded on glass coverslips in 4-well dishes. Then the apoptosis of cells was analyzed via TUNEL assay using Cell Death Detection Kit (Roche Diagnostics) following the manufacturer’s recommended procedure. The samples were stained with DAPI to visualize total cells. TUNEL-positive cells were counted and normalized to total MIN6 cells.

### Edu-594 Staining

Cell proliferation was assessed by Edu-594 staining. MIN6 cells were seeded on glass coverslips in 4-well dishes. Then cells were fixed with 10% formalin for 25 min and then permeabilized in PBS containing 0.2% Triton X-100 for 5 min at room temperature. The cells were blocked using 5% protease-free BSA in PBS for 30 min at room temperature followed by incubation with anti-Edu-594 primary antibody (1:50, Proteintech) at 4°C overnight and then with secondary antibody for 1 h, and subsequently stained with DAPI for 5 min at room temperature before detection by fluorescence microscopy.

### MTT Assay

An MTT kit (Roche Diagnostics, Indianapolis, IN, USA) was used to determine the proportion of viable MIN6 cells. 5 × 10^3^ cells were seeded into 12-well plates. MTT (500 µg/ml) was added to each well for 3 h at 37°C. MTT reagent was removed and dimethyl sulfoxide was added to dissolve the cells. The cells were kept in the dark and the optical density was read at 570 nm using a microplate reader. Experiments were repeated three times.

### Glucose-Stimulated Insulin Secretion (GSIS)

Min6 cells were seeded in 96-well plates. Then cells were incubated with Krebs-Ringer bicarbonate HEPES (KRBH, PanEra, Guangzhou, China) buffer for 1 h at 37°C. Thereafter, the KRBH buffer was removed and 1 ml KRBH containing 5.0 or 20.0 mM glucose was added into subgroups separately for 1 h at 37°C. The insulin levels were determined using an ELISA Kit for insulin (Cloud-Clone Corp., Wuhan, China) according to the manufacturer’s instructions.

### Immunofluorescence

After formalin fixation and paraffin embedding, the pancreatic tissues were cut into sections (4 μm). Pancreas sections were immunostained for insulin (1:50, Proteintech). Pancreatic islet proliferation was identified by staining sections with anti-Ki-67 polyclonal antibody (1:50, Proteintech). Cell nuclei were counterstained with DAPI.

### Statistical Analysis

Statistical analysis was performed using SPSS version 16.0 software. One-way ANOVA was utilized to compare the difference between the groups. A *P*-value less than 0.05 was considered statistically significant.

## Results

### Cell Experiment

#### The Expression of Metrnl

**FIGURE 2:**
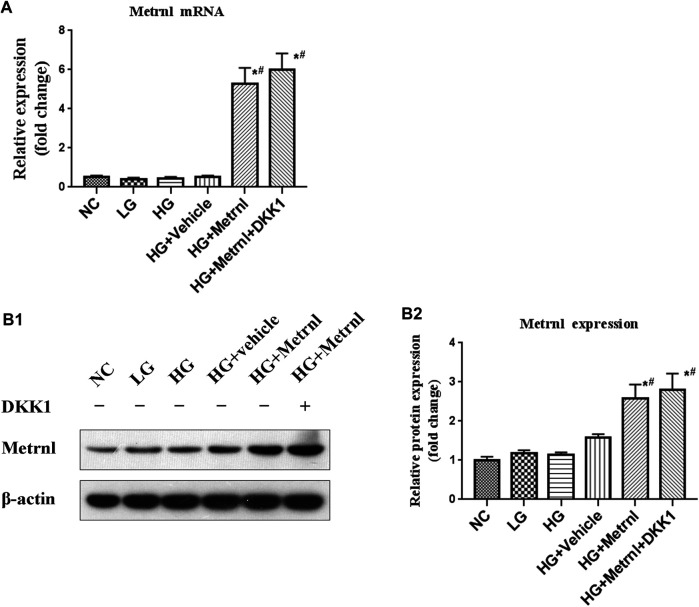
The expression of Metrnl after pCDH-Metrnl vector transfection. **(A)** The gene expression of Metrnl quantified by real-time PCR. Data are presented as the fold change of transcripts for the target gene in the indicated groups normalized to β-actin. **(B)** Representative western blot showing Metrnl protein levels in MIN6 cells of different groups. Band densities were converted to a bar graph. All data are expressed as mean ± SEM (*n* = 8/group [mRNA], *n* = 8/group [protein]). *p < 0.05 for differences vs. normal control group; ^#^ *p* < 0.05 for differences vs. HG group. NC: normal control, LG: low glucose, HG: high glucose, DKK1: dickkopf 1.

Metrnl and DKK1 groups showed higher mRNA and protein expression of Metrnl compared with the other groups (Figure 2). This confirmed the successful transfection of Metrnl.

### β Cell Apoptosis

**FIGURE 3:**
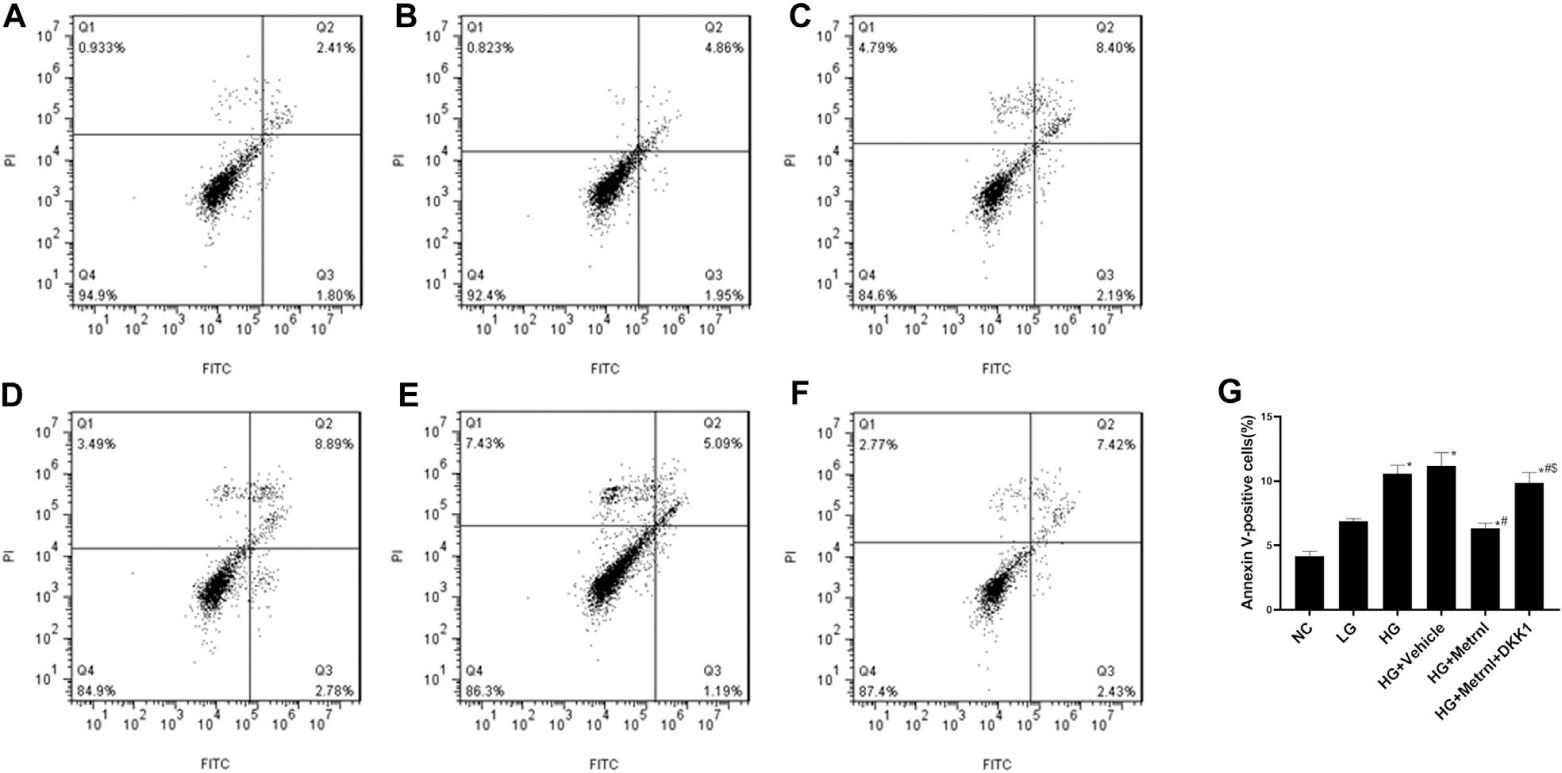
β cell apoptosis between different groups evaluated using flow cytometry analysis. **(A)** normal control (NC); **(B)** low glucose (LG); **(C)** high glucose (HG); **(D)** HG + Vehicle; **(E)** HG + Metrnl; **(F)** HG + Metrnl + DKK1. **(G)** quantitative analysis of different groups.

**FIGURE 4:**
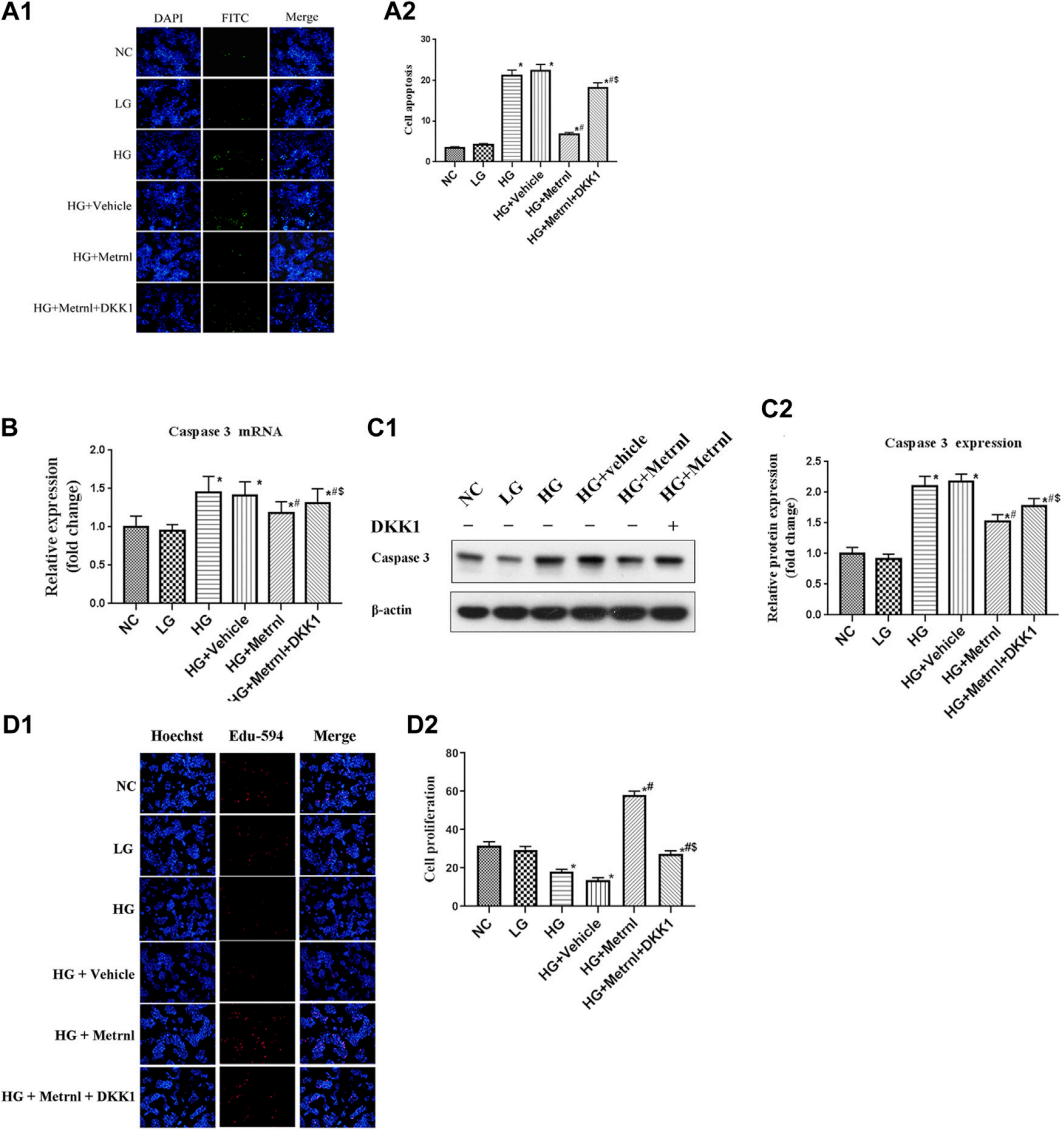
**(A)** β cell apoptosis between different groups assessed by TUNEL assay. NC: normal control, LG: low glucose, HG: high glucose, DKK1: dickkopf 1. **(B)** The gene expression of caspase 3 was quantified by real-time PCR. Data are presented as the fold change of transcripts for the target gene in the indicated groups normalized to β-actin. All data are expressed as mean ± SEM [*n* = 8/group (mRNA)]. **(C)** Representative western blot showing caspase 3 protein levels in MIN6 cells of different groups. Band densities were converted to a bar graph. All data are expressed as mean ± SEM [*n* = 8/group (protein)]. **(D)** β cell proliferation evaluated using Edu-594 staining between different groups. *p < 0.05 for differences vs. normal control group; ^#^ *p* < 0.05 for differences vs. HG group; ^$^ *p* < 0.05 for differences vs. HG + Metrnl group. NC: normal control, LG: low glucose, HG: high glucose, DKK1: dickkopf 1.

As shown in Figures 3 and 4A, flow cytometry analysis and TUNEL assay both showed that the β cell apoptosis in the high glucose group was higher than in the normal control group. Then we detected the mRNA and protein expression of caspase 3 in different groups and found that high glucose could induce the mRNA and protein expression of caspase 3 (Figures 4B,C). In addition, Metrnl treatment ameliorated the elevated β cell apoptosis and caspase 3 expression induced by high glucose. This demonstrates that Metrnl has a protecting role against β cell apoptosis.

### β Cell Proliferation

**FIGURE 5:**
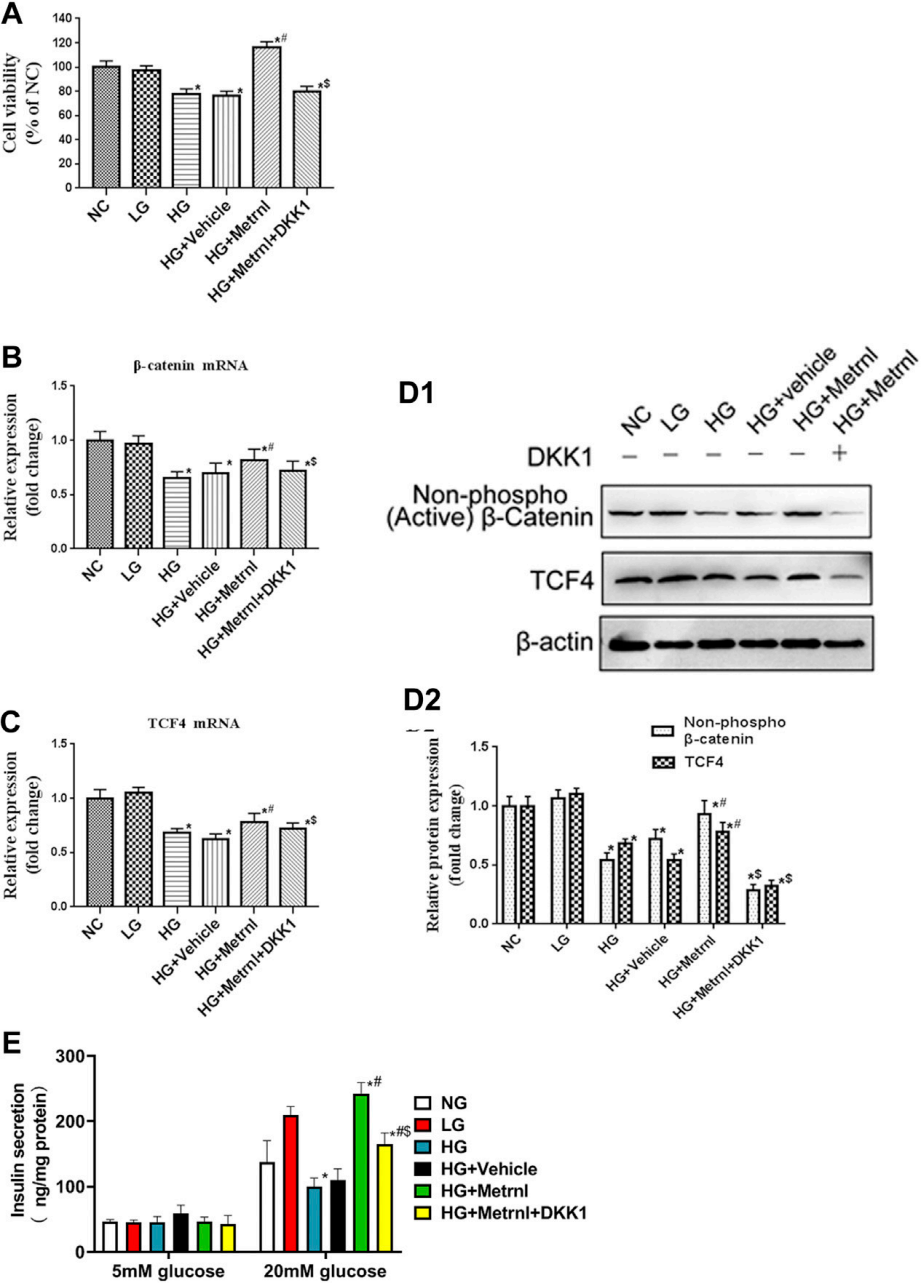
**(A)** Cell viability evaluated using MTT assay between different groups. **(B)** The gene expression of β-catenin was quantified by real-time PCR. Data are presented as the fold change of transcripts for the target gene in the indicated groups normalized to β-actin. **(C)** The gene expression of TCF4 was quantified by real-time PCR. Data are presented as the fold change of transcripts for the target gene in the indicated groups normalized to β-actin. All data are expressed as mean ± SEM (*n* = 8/group [mRNA]). **(D)** Representative western blot showing β-catenin and TCF4 protein levels in MIN6 cells of different groups. Band densities were converted to a bar graph. All data are expressed as mean ± SEM [*n* = 8/group (protein)]. **(E)** glucose-stimulated insulin secretion (GSIS) test in MIN6 cells of different groups.

Immunofluorescence results showed that the β cell proliferation was significantly decreased after high glucose incubation (Figure 4D). Moreover, the cell viability was significantly decreased in the high glucose group than in the normal control group (Figure 5A). The β cell proliferation and cell viability were significantly increased after Metrnl treatment (Figures 4D and 5A). We got a conclusion that Metrnl could promote the β cell proliferation.

### WNT/β-Catenin Pathway

WNT/β-catenin pathway is correlated with the cell apoptosis and proliferation. As shown in Figures 5B–D, high glucose contributed to decreased mRNA and protein expression of β-catenin and TCF4 which are the markers of WNT/β-catenin pathway. Decreased WNT/β-catenin pathway led to promoted apoptosis and inhibited proliferation of β cell. Metrnl treatment contributed to increased mRNA and protein expression of β-catenin and TCF4. This indicates that Metrnl could inhibit β cell apoptosis and promote β cell proliferation via WNT/β-catenin pathway.

### GSIS Analysis

As shown in Figure 5E, Metrnl overexpression promoted insulin secretion under high-glucose stimulation, and high glucose inhibited insulin secretion under high-glucose stimulation. There were no effects of Metrnl overexpressionon on the insulin secretion under basal-glucose stimulation.

### Wnt Antagonist

As WNT/β-catenin pathway is activated by Metrnl, we used WNT antagonist DKK1 to inhibit WNT/β-catenin pathway. We then determined the effects of Metrnl on the apoptosis and proliferation of β cell after suppressing WNT/β-catenin pathway. Treatment with Dickkopf 1 partly restored reduced apoptosis and promoted proliferation of β cell that were due to Metrnl treatment. This confirms that Metrnl play a role in regulating β cell function by promoting WNT/β-catenin pathway.

### Animal Experiment

#### Body Weight and Blood Glucose

**FIGURE 6:**
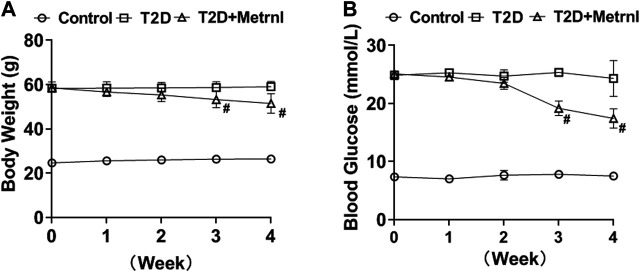
The body weight and blood glucose in mice. **(A)** The body weight differences of mice between three groups. **(B)** The blood glucose differences between three groups. All data are expressed as mean ± SEM(*n* = 8/group). ^#^ *p* < 0.05 for differences vs. T2D group. T2D: type 2 diabetes.

Mice in T2D group showed higher body weight and blood glucose compared with the controls (Figure 6). Metrnl treatment alleviated the increased body weight and blood glucose of T2D mice from the third week (Figure 6).

### The Expression of Metrnl in pancreatic Tissues

**FIGURE 7:**
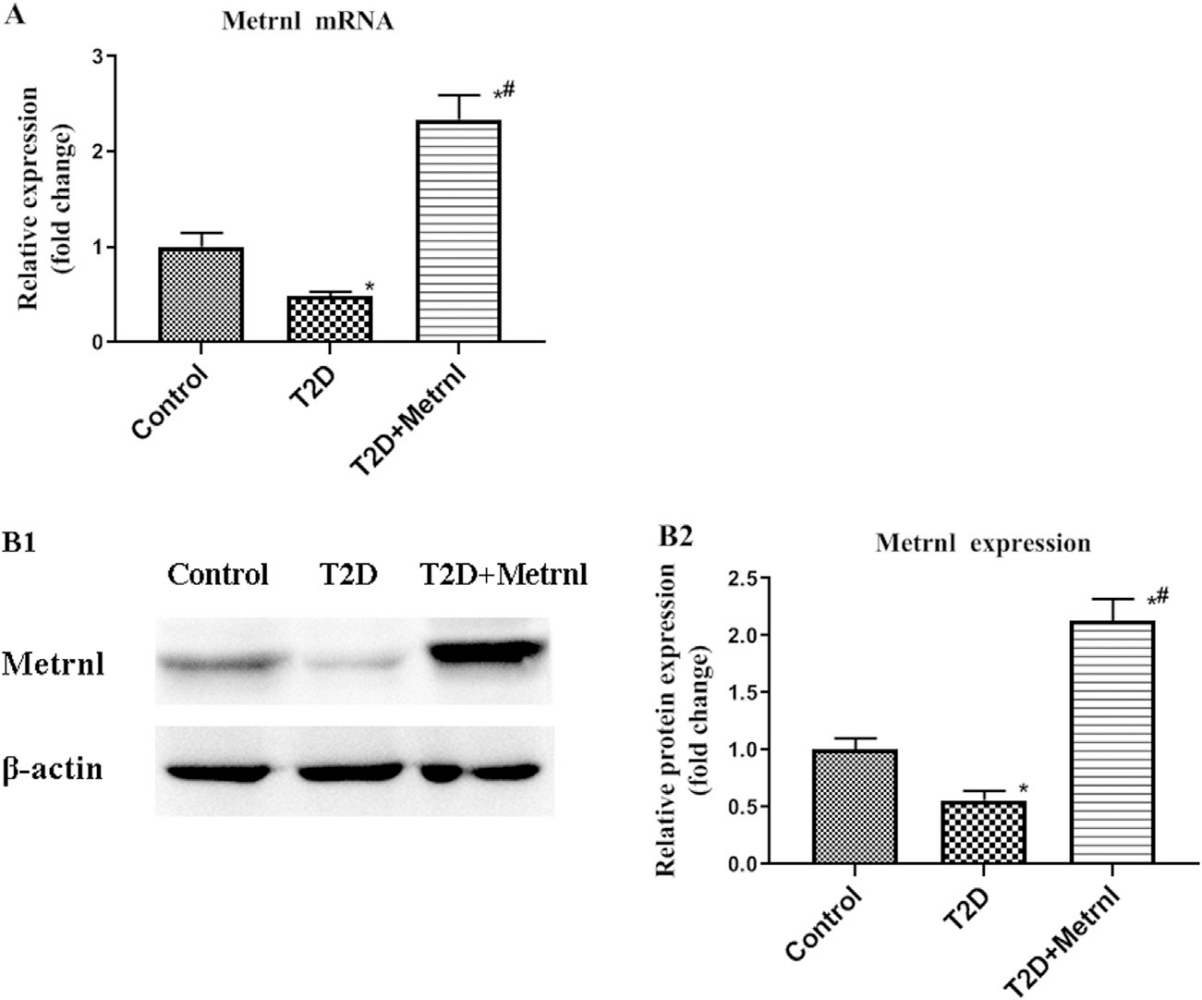
The expression of Metrnl in pancreatic tissues of mice. **(A)** The gene expression of Metrnl quantified by real-time PCR. Data are presented as the fold change of transcripts for the target gene in the indicated groups normalized to β-actin. **(B)** Representative western blot showing Metrnl protein levels in MIN6 cells of different groups. Band densities were converted to a bar graph. All data are expressed as mean ± SEM [*n* = 8/group (mRNA), *n* = 8/group (protein)]. *p < 0.05 for differences vs. control group; ^#^ *p* < 0.05 for differences vs. T2D group. T2D: type 2 diabetes.

Decreased mRNA and protein expression of Metrnl was found in the pancreatic tissues of T2D mice compared with the control group (Figure 7). In addition, T2D+Metrnl group showed higher mRNA and protein expression of Metrnl compared with the other two groups (Figure 7).

### β Cell Apoptosis

**FIGURE 8:**
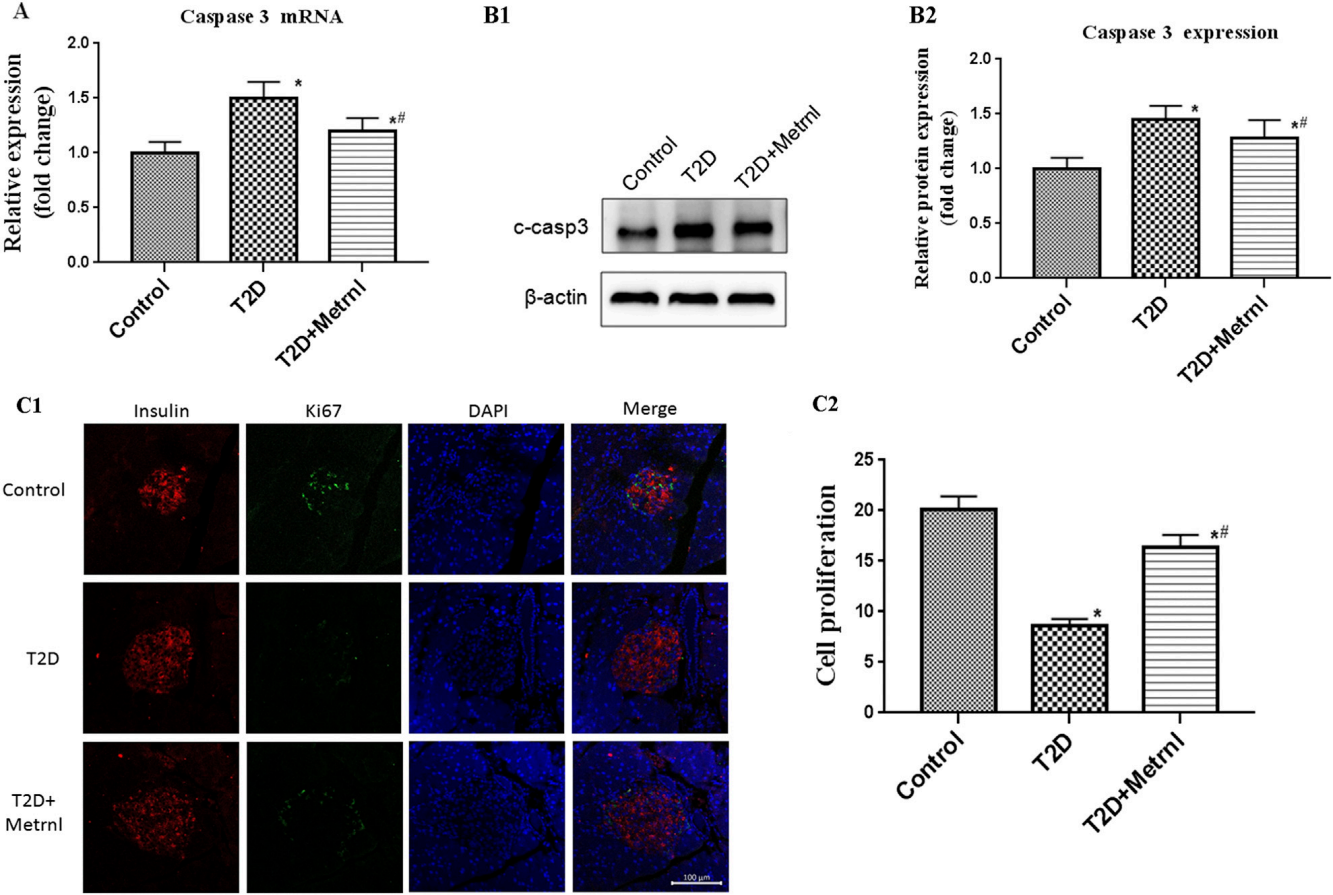
**(A)** The gene expression of caspase 3 was quantified by real-time PCR. Data are presented as the fold change of transcripts for the target gene in the indicated groups normalized to β-actin. All data are expressed as mean ± SEM [*n* = 8/group (mRNA)]. **(B)** Representative western blot showing caspase 3 protein levels in the pancreatic tissue of mice. Band densities were converted to a bar graph. All data are expressed as mean ± SEM [*n* = 8/group (protein)]. **(C)** β cell proliferation in the pancreatic tissue of mice evaluated using Ki67 and insulin staining. All data are expressed as mean ± SEM(*n* = 8/group). ^*^ *p* < 0.05 for differences vs. control group; ^#^ *p* < 0.05 for differences vs. T2D group. T2D: type 2 diabetes.

As shown in Figures 8A,B, the expression of cleaved caspase 3 in the pancreatic tissues of T2D mice was significantly increased than in the control mice. Caspase 3 expression was suppressed by Metrnl treatment. This indicates that Metrnl has a protecting role against β cell apoptosis which is assessed using caspase-3 expression.

### β Cell Proliferation

Immunofluorescence using Ki67 and insulin antibody were used to assess the β cell proliferation. The β cell proliferation is decreased in T2D mice than in the control mice (Figure 8C). In addition, Metrnl group showed significantly higher β cell proliferation compared with T2D group (Figure 8C).

### WNT/β-Catenin Pathway

**FIGURE 9:**
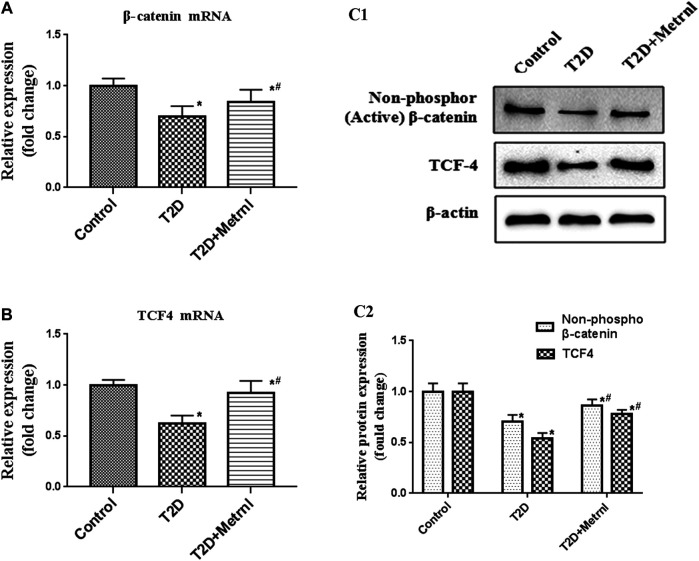
The expression of the marker proteins of WNT/β-catenin pathway in the pancreatic tissue of mice quantified by real-time PCR and western blot. **(A)** The gene expression of β-catenin was quantified by real-time PCR. Data are presented as the fold change of transcripts for the target gene in the indicated groups normalized to β-actin. **(B)** The gene expression of TCF4 was quantified by real-time PCR. Data are presented as the fold change of transcripts for the target gene in the indicated groups normalized to β-actin. **(C)** Representative western blot showing β-catenin and TCF4 protein levels in the pancreatic tissue of mice. Band densities were converted to a bar graph. All data are expressed as mean ± SEM [*n* = 8/group (mRNA), *n* = 8/group (protein)]. *p < 0.05 for differences vs. control group; ^#^ *p* < 0.05 for differences vs. T2D group. T2D: type 2 diabetes.

As shown in Figure 9, the expression of the marker proteins of WNT/β-catenin pathway including β-catenin and TCF4 were decreased in the pancreatic tissues of T2D mice than in the control mice. Metrnl group showed significantly higher expression of β-catenin and TCF4 compared with T2D group.

## Discussion

Metrnl resumed the decreased insulin secretion both in palmitate-incubated murine myoblasts cells and the skeletal muscle from high-fat diet-fed mice (Jung et al., 2018). Furthermore, Glucose intolerance was alleviated after Metrnl adminstration in mice (Jung et al., 2018). Metrnl-treated obese mice showed improved glucose tolerance during intraperitoneal glucose tolerance test (IPGTT) (Rao et al., 2014). Lee reported that Metrnl increased glucose uptake in the skeletal muscle cells and increased the phosphorylation of transcriptional repressor of glucose transporter 4 (Lee et al., 2020). In addition, Metrnl injection alleviated glucose tolerance status in mice with high-fat-diet-induced obesity or T2D (Lee et al., 2020). Sun Y reported that intravenous administration of metrnl reduced blood glucose levels and the area under the curve of IPGTT in NOD mice (Yao et al., 2020). Our results showed that Metrnl improved blood glucose in db/db mice. These results indicate that Metrnl may serve as a regulator of glucose metabolism and diabetes.

Recent studies showed the correlation between serum Metrnl and diabetes. Serum Metrnl levels were found to be lower in T2D patients compared with the healthy controls (Dadmanesh et al., 2018; Lee et al., 2018; El-Ashmawy et al., 2019; Fadaei et al., 2020). Serum Metrnl was negatively correlated with the blood glucose, fasting insulin, and HbA1c (Fadaei et al., 2020; Jung et al., 2018). Serum Metrnl is correlated with diabetes and may be utilized as a new biomarker for diabetes diagnosis.

Skeletal muscle, the largest organ in the human body, plays a great role in maintaining total metabolic homeostasis. Recent studies show that skeletal muscle secretes a variety of myokines which are involved in the metabolic and inflammatory processes. Myokines such as fibroblast growth factor 21 and irisin were found to be correlated with β cell function. Fibroblast growth factor 21 protected against lipotoxicity-induced β cell dysfunction and apoptosis (Pedersen and Edward, 2009). Irisin injection promoted β cell survival and enhanced insulin secretion in mice (Natalicchio et al., 2017). Our study also indicated that Metrnl could inhibit β cell apoptosis and activate β cell proliferation in mouse pancreatic β cells and db/db mice. Metrnl may be a promising therapeutic candidate for T2D.

Wnt/β-catenin signal pathway plays a critical role in cellular proliferation (MacDonald et al., 2009). Activation of the β-catenin partially protected against H_2_O_2_-induced apoptosis of islet microvascular endothelium cell (Wang et al., 2017). Dioscin promoted the β cells proliferation via Wnt/β-Catenin signaling pathways (Yu et al., 2018). Yao reported that geniposide promoted β cell proliferation and inhibited β cell apoptosis in cultured mouse islets through activating Wnt signaling (Yao et al., 2015). We demonstrated that Metrnl could inhibit β cell apoptosis and activate β cell proliferation via promoting WNT/β-catenin pathway. These results point to the key role of WNT/β-catenin pathway in maintaining β cell function.

The present study showed that Metrnl treatment led to decreased body weight in mice. Jung also reported that Metrnl administration reduced high-fat diet-induced body weight gain in mice (Jung et al., 2018). This indicates the potential role of Metrnl in obesity. Metrnl inhibited human adipocyte differentiation which is assessed using decreased lipogenesis and adipogenesis (Löffler et al., 2017). Metrnl stimulated energy expenditure and promoted the expression of genes associated with beige fat thermogenesis (Rao et al., 2014). Serum Metrnl levels were decreased in obese patients with T2D than in lean T2D patients (Fadaei et al., 2020). Patients with obesity showed lower Metrnl levels compared to controls. And Metrnl levels were significantly increased in 6 and 12 months after laparoscopic sleeve gastrectomy (Pellitero et al., 2018).

In conclusion, Metrnl ameliorates β cell function by inhibiting β cell apoptosis of and promoting β cell proliferation via activating the WNT/β-catenin pathway.

## Data Availability

The raw data supporting the conclusions of this article will be made available by the authors, without undue reservation.
